# Supplementary material for: Shared requirement for MYC upstream super-enhancer region in tissue regeneration and cancer
Source: Life Sci Alliance. 2025 Apr 3;8(6):e202403090. doi: 10.26508/lsa.202403090 (PMC11969384; doi:10.26508/lsa.202403090)
Supplement: Supplementary file 7 [file LSA-2024-03090_SdataF5.pdf]

Source data Fig 5B

Cell type	Genotype	Number	% of total cells
$SSC1_F$	WT	898	13,405
$SSC2_F$	WT	69	1,030
$SSC3_F$	WT	601	8,971
$SSC4_F$	WT	78	1,164
$SSC5_F$	WT	40	0,597
TA	WT	2453	36,617
EP	WT	1150	17,167
EC	WT	208	3,105
EE	WT	200	2,986
GC	WT	772	11,524
FB	WT	230	3,433

Cell type	Genotype	Number	% of total cells
$SSC1_F$	$Myc^{\Delta 2\text{-}540/\Delta 2\text{-}540}$	52	1,34
$SSC2_F$	$Myc^{\Delta 2\text{-}540/\Delta 2\text{-}540}$	663	17,09
$SSC3_F$	$Myc^{\Delta 2\text{-}540/\Delta 2\text{-}540}$	54	1,39
$SSC4_F$	$Myc^{\Delta 2\text{-}540/\Delta 2\text{-}540}$	454	11,70
$SSC5_F$	$Myc^{\Delta 2\text{-}540/\Delta 2\text{-}540}$	345	8,89
TA	$Myc^{\Delta 2\text{-}540/\Delta 2\text{-}540}$	525	13,53
EP	$Myc^{\Delta 2\text{-}540/\Delta 2\text{-}540}$	402	10,36
EC	$Myc^{\Delta 2\text{-}540/\Delta 2\text{-}540}$	1018	26,24
EE	$Myc^{\Delta 2\text{-}540/\Delta 2\text{-}540}$	41	1,06
GC	$Myc^{\Delta 2\text{-}540/\Delta 2\text{-}540}$	237	6,11
FB	$Myc^{\Delta 2\text{-}540/\Delta 2\text{-}540}$	88	2,27
