# Supplementary material for: Shared requirement for MYC upstream super-enhancer region in tissue regeneration and cancer
Source: Life Sci Alliance. 2025 Apr 3;8(6):e202403090. doi: 10.26508/lsa.202403090 (PMC11969384; doi:10.26508/lsa.202403090)
Supplement: Supplementary file 10 [file LSA-2024-03090_SdataF6.pdf]

Source data Fig 6A

Survival of mice (Days)		
Apc$^{Min/+}$; Myc$^{Δ2\text{-}540/Δ2\text{-}540}$	***Apc$^{Min/+}$***	***Apc$^{Min/+}$; Myc-335-/-***
500	167	370
541	156	311
626	156	353
626	156	306
633	134	260
524	134	277
643	171	277
631	161	
572	132	
572		
422		
550		
505		
540		
553		
510		
464		
219		

Source data Fig 6B

**Genotype**	**Cell type**	**Total Nr of cells**	**Ly6a (Sca1)$^+$**	**% of Ly6a (Sca1)$^+$ cells**
Apc$^{Min/+}$	SC	1249	36	2,882
Apc$^{Min/+}$; Myc$^{Δ2\text{-}540/Δ2\text{-}540}$	SC	3431	40	1,166
Apc$^{Min/+}$	TA	1435	59	4,111
Apc$^{Min/+}$; Myc$^{Δ2\text{-}540/Δ2\text{-}540}$	TA	3784	60	1,586
Apc$^{Min/+}$	EP	1067	170	15,933
Apc$^{Min/+}$; Myc$^{Δ2\text{-}540/Δ2\text{-}540}$	EP	1896	62	3,270

Source data Fig 6F

**Genotype**	**Nr of stem cells**	**Nr of SSC$_{reg}$ cells**	**% of SSC$_{reg}$ cells**
WT	1578	5	0,316856781
Myc$^{Δ2\text{-}540/Δ2\text{-}540}$	1339	4	0,298730396
WT$_{IR}$ (2 dpi)	308	89	28,8961039
Myc$^{Δ2\text{-}540/Δ2\text{-}540}$$_{IR}$ (2dpi)	714	69	9,663865546
Apc$^{Min/+}$	1249	103	8,246597278
Apc$^{Min/+}$; Myc$^{Δ2\text{-}540/Δ2\text{-}540}$	3431	32	0,93267269
