# Supplementary material for: Shared requirement for MYC upstream super-enhancer region in tissue regeneration and cancer
Source: Life Sci Alliance. 2025 Apr 3;8(6):e202403090. doi: 10.26508/lsa.202403090 (PMC11969384; doi:10.26508/lsa.202403090)
Supplement: Supplementary file 11 [file LSA-2024-03090_SdataFS6.pdf]

Source data Supplementary Fig S6A

Survival of mice (Months)	
Pten$^{+/-}$	*Pten*$^{+/-}$; *Myc*$^{\Delta 2\text{-}540/\Delta 2\text{-}540}$
8	2
10	7
10	6
5	4
9	7
9	9
4	5
11	7
5	7
9	10
5	8
6	5
6	9
5	9
10	6
6	6
9	10
6	8
6	10
	8
	8
	9
	7
	7
	6
	6
	8
	9
